# Supreme Court and the Practice of Oncology

**DOI:** 10.1093/oncolo/oyac111

**Published:** 2022-06-08

**Authors:** Bruce A Chabner, Susan E Bates

## Abstract

The past and future Editors-in-Chief of The Oncologist offer thoughts on the recent U.S. Supreme Court leak and its significance for other areas of medical practice.

As the past and future Editors-in-Chief of *The Oncologist*, we wish to offer our thoughts on the leaked US Supreme Court draft opinion in a pending case, *Dobbs v. Jackson Women’s Health Organization*, supporting the decision to overturn *Roe v. Wade*. As a draft, it is not the court’s final opinion, but it appears that a majority has determined that the decision about whether and when a woman has the right to terminate her pregnancy belongs in the hands of the states and is not a constitutional right of US citizens. In the draft opinion, Justice Alito reasoned that the right to have an abortion was not explicitly granted in the Constitution. It should be acknowledged that at the time the Constitution was written, and for 100 years afterward, there was no known way to perform a safe abortion. Anesthesia and sterile procedures were yet to be discovered in medical practice, and many patients died in attempting to end their pregnancies. To grant this right to women at the time the Constitution was written would have been impossible.

If the draft opinion becomes final, abortion restrictions would go into effect in nearly half of the states, and in 13 states, would activate post-*Roe* “trigger” laws that ban all or nearly all abortions. In most states, abortions to save the life of the mother would be allowed, but it is unclear what will be considered a “life-saving” situation. Would “life-saving” apply only to situations in which the life of the mother is in imminent danger if the pregnancy continues (eclampsia, hemorrhage), or would it also include cases in which a delay in treatment could adversely affect the individual’s survival, as for example a delay in adjuvant therapy for breast cancer or a delay in the treatment of highly aggressive cancers or leukemias? In some states, pending legislation would not only ban abortion, but would also subject the mother and the doctors to prosecution for homicide if an abortion was performed. Contrary to the draft report’s reasoning, in our medical opinion, the right to undergo an abortion should be a constitutional right, and the decision made by the pregnant individual alone.

The decision to end a pregnancy will always be a difficult one, fraught with religious and ethical implications. And medically indicated abortions are as tragic as any other. This decision requires an extended and thoughtful discussion, with the expectant mother at the center of the deliberation, and a balancing of multiple considerations in each unique case.

A Supreme Court judgment against abortion portends a serious threat to the rational practice of medicine. It establishes a precedent for intrusion into other areas of medical practice. Oncologists clearly have standing in this debate. Stem cell research, in vitro fertilization, genome-altering therapies, contraception, and other categories of research and practice relevant to cancer treatment have evoked political debate and could become the next issue before the Supreme Court. Physicians, whether individually or through academic or professional organizations, need to ensure that the consequences of such legal actions are fully understood and considered.

## Conflict of Interest

**Bruce A. Chabner:** PharmaMar, EMD Serono, Cyteir, Eli Lilly & Co., Chugai Pharmaceuticals, Takeda (C/A), Cyteir (H), Biomarin, Seattle Genetics, GlaxoSmithKline, PharmaMar, Loxo, Blueprint, Immunomedics, Constellation, Bluebird, Alnylam, SpringWorks, Forty Seven (OI), Eli Lilly & Co., Genentech (ET); **Susan E. Bates:** None.

(C/A) Consulting/advisory relationship; (RF) Research funding; (E) Employment; (ET) Expert testimony; (H) Honoraria received; (OI) Ownership interests; (IP) Intellectual property rights/inventor/patent holder; (SAB) Scientific advisory board.

